# The Debate on Cancer of the Breast

**Published:** 1898-03-05

**Authors:** 


### THE DEBATE ON CANCER OF THE BREAST.

The debate which has occupied the last three meetings of the Royal Medical and Chirurgical Society has been very instructive, although, as was to be expected, its results have been indefinite. Probably not one of the surgeons who took part has been led to alter his opinion; some, indeed, may have had doubts removed, and have even been confirmed in their mode of practice by finding how largely their "heresies" are shared by men of standing. Nevertheless, to the great outside professional public, whose function it is to advise the lay public, some very useful lessons have been taught, more particularly in the direction of hopefulness.

The old text-book view, that a patient suffering from cancer is doomed, and that the man who holds out a hope of cure is a charlatan and a quack, has been thoroughly routed. Of course, the old controversy about the meaning of words remains, and we seem as far as ever from deciding what is meant by a cure. But if we take as a basis the statement made that 40 or 50 per cent. of the cases which have undergone the larger operation have been successful, *i.e.*, have remained free for three years, and that made by Professor Watson Cheyne that, of his own cases, there had been, so far as he knew, no recurrences in those that had remained free from the disease for that period, we cannot but feel how enormously the point of view from which cancer of the breast is to be regarded has changed since the days when those medical men were educated who are now but middle-aged. What has to be recognised is that, although cancer of the breast is a disease which is so variable in its progress that in regard to individuals it is always unsafe to prophesy, the chances of prolongation of life, and of death taking place from some other malady, are so great that, speaking always in averages, one may now properly urge, not merely that operation is the only thing to be done, but that it can be done with very good hope of a successful result.

It was, indeed, admitted that, after the larger operations now being performed, internal deposits were found with greater frequency than before, but it was pointed out—and we think justly—that this was the natural consequence of patients having been saved from the local recurrences, and the still earlier death which would have been caused by them.

There was much difference of opinion as to the extent of operation which ought to be undertaken, and as to the comparative advantages of the "old" and the Heidenhain-Stiles operation; and, in view of the apparent capriciousness of the progress of cancer, and the strikingly good results which sometimes follow much smaller operations than those which are now so commonly advocated, several surgeons expressed the feeling that as time went on the new operation would take a less prominent place. There is one point, however, in regard to which all the speakers were agreed—viz., the necessity of early removal, and this is what should be firmly and persistently impressed, not only on general practitioners, but perhaps even on the public; for the greater the chance of our being able to give permanent relief—whether we call it cure or not—the greater is the urgency of not losing a moment in submitting the patient to operation, for literally every moment is of importance. Perhaps we may without offence also suggest that another fact worthy of being remembered is that the modern extensions of the operation have entirely removed amputation of the breast from the category of small operations. It is a large operation in every sense, and not only the ultimate success, but the life of the patient at the time, depend on the perfect carrying out of many details which are not always easy of arrangement, except to those who are constantly engaged in surgical work. "Specialism again!" some will say; but it cannot be helped.

